# Harmonising data on the correlates of physical activity and sedentary behaviour in young people: Methods and lessons learnt from the international Children’s Accelerometry database (ICAD)

**DOI:** 10.1186/s12966-017-0631-7

**Published:** 2017-12-20

**Authors:** Andrew J. Atkin, Stuart J. H. Biddle, Stephanie T. Broyles, Mai Chinapaw, Ulf Ekelund, Dale W. Esliger, Bjorge H. Hansen, Susi Kriemler, Jardena J. Puder, Lauren B. Sherar, Esther M. F. van Sluijs, On behalf of the International Children’s Accelerometry Database (ICAD) Collaborators, L. B. Andersen, S. Anderssen, A. J. Atkin, G. Cardon, R. Davey, U. Ekelund, D. W. Esliger, P. Hallal, B. H. Hansen, K. F. Janz, S. Kriemler, N. Møller, L. Molloy, A. Page, R. Pate, J. J. Puder, J. Reilly, J. Salmon, L. B. Sardinha, L. B. Sherar, A. Timperio, E. M. F. van Sluijs

**Affiliations:** 1 School of Health Sciences, Faculty of Medicine and Health Sciences, University of East Anglia, Norwich Research Park, Norwich, NR4 7TJ UK; 2 MRC Epidemiology Unit and UKCRC Centre for Diet and Activity Research (CEDAR), University of Cambridge School of Clinical Medicine, Institute of Metabolic Science, Cambridge Biomedical Campus, Cambridge, UK; 3 Institute for Resilient Regions, University of Southern Queensland, Springfield, QLD Australia; 4 Pennington Biomedical Research Center, Baton Rouge, LA USA; 5 Department of Public and Occupational Health, Amsterdam Public Health research institute, VU University Medical Center, Amsterdam, Netherlands; 6 Department of Sport Science, Norwegian School of Sport Sciences, Oslo, Norway; 7 National Centre for Sport and Exercise Medicine, School of Sport, Exercise and Health SciencesLoughborough University, Loughborough, UK; 8 Epidemiology, Biostatistics and Prevention Institute, University of Zurich, Zurich, Switzerland; 9 Service of Endocrinology, Diabetes and Metabolism & Service of Pediatric Endocrinology, Diabetology and Obesity, Lausanne University Hospital, Lausanne, Switzerland

**Keywords:** Physical activity, Sedentary behaviour, Children, Adolescents, Retrospective harmonisation, Data pooling, ICAD

## Abstract

**Background:**

Large, heterogeneous datasets are required to enhance understanding of the multi-level influences on children’s physical activity and sedentary behaviour. One route to achieving this is through the pooling and co-analysis of data from multiple studies. Where this approach is used, transparency of the methodology for data collation and harmonisation is essential to enable appropriate analysis and interpretation of the derived data. In this paper, we describe the acquisition, management and harmonisation of non-accelerometer data in a project to expand the International Children’s Accelerometry Database (ICAD).

**Method:**

Following a consultation process, ICAD partners were requested to share accelerometer data and information on selected behavioural, social, environmental and health-related constructs. All data were collated into a single repository for cataloguing and harmonisation. Harmonised variables were derived iteratively, with input from the ICAD investigators and a panel of invited experts. Extensive documentation, describing the source data and harmonisation procedure, was prepared and made available through the ICAD website.

**Results:**

Work to expand ICAD has increased the number of studies with longitudinal accelerometer data, and expanded the breadth of behavioural, social and environmental characteristics that can be used as exposure variables. A set of core harmonised variables, including parent education, ethnicity, school travel mode/duration and car ownership, were derived for use by the research community. Guidance documents and facilities to enable the creation of new harmonised variables were also devised and made available to ICAD users. An expanded ICAD database was made available in May 2017.

**Conclusion:**

The project to expand ICAD further demonstrates the feasibility of pooling data on physical activity, sedentary behaviour and potential determinants from multiple studies. Key to this process is the rigorous conduct and reporting of retrospective data harmonisation, which is essential to the appropriate analysis and interpretation of derived data. These documents, made available through the ICAD website, may also serve as a guide to others undertaking similar projects.

## Background

Much has been written of the need for very large-scale studies in contemporary epidemiology and public health. [1–4] Often the discussion is framed in the context of genomic research and the exploration of gene-environment interactions, but many branches of science, including behavioural epidemiology, will benefit from having data on very large numbers of participants. Larger samples typically increase exposure heterogeneity and enhance our ability to explore complex interactions (effect modification) amongst (multi-level) exposures. These qualities are pertinent to the study of physical activity and sedentary behaviour in young people, considered as either determinants of health (exposure) or as targets for behaviour change interventions (outcome). Relative to the adult population, heterogeneity in anthropometric and cardiometabolic health markers is reduced in young people, thus large samples are required to identify the small, but potentially important, associations with components of physical activity. [5, 6] Moreover, current understanding of the determinants of physical activity and sedentary behaviour is limited by a reliance on single-country, relatively small studies that lack exposure heterogeneity and the statistical power required to explore interactions amongst factors from different levels of the ecological model. [7, 8]

One response to the need for larger-scale epidemiological studies has been to establish new cohorts, such as UK Biobank [9, 10] or the Kadoorie Biobank [11, 12], which have collected detailed genetic and phenotypic information on many thousands of participants and followed them over time. As these resources mature they will provide invaluable scientific insight into a range of complex outcomes, but such studies require significant financial investment, are logistically complex and limited in scope by the need to manage participant burden. Moreover, most of these new cohorts focus exclusively on the adult population, with efforts to conduct studies of a similar scale in young people, through the formation of new birth cohorts for example, proving to be extremely challenging. [13, 14] An alternative approach has been to establish multiple smaller, geographically diverse cohorts in parallel, with each study site using a common methodology, either in its entirety or within specific topic areas. [15–17] This approach serves to limit the burden within each study centre and increases sample heterogeneity but requires consensus amongst collaborators regarding methodology and, again, requires significant financial investment to support data collection. A common limitation of both strategies outlined above is that it can take many years for new cohorts to mature and realise their potential through longitudinal data on both exposures and outcomes. A third option is to combine information from existing studies, sometimes referred to as data pooling. This strategy seeks to maximise heterogeneity and statistical power by combining data from selected studies in such a way that enables simultaneous analysis through one- or two-stage individual participant meta-analysis, details of which can be found elsewhere. [18] It offers a route to meeting the demands of contemporary epidemiology in a shorter timeframe than that needed to establish a new cohort and with reduced financial and logistical demands relative to primary data collection. It also serves to maximise funders’ return on their investments through better use of existing data. Data pooling has been widely employed in some fields of research [19] but has been used less frequently in the physical activity domain, particularly with young people. [20, 21]

A growing body of literature is emerging to address the myriad legal and methodological challenges presented by pooling data across studies, much of which has emanated from the Maelstrom Research collaboration. [19, 22–29] A key challenge lies in the administration and management of data from multiple studies, the complexity of which will vary depending upon whether the data are physically relocated to a central repository, for example, or retained within the host institution. Perhaps the most frequently discussed consideration relevant to data pooling, however, is the derivation of analytical variables that are comparable, or at least more comparable, across contributing studies, a process known as data harmonisation. [19, 21, 27, 30] Central to the harmonisation process is a judgement on whether data from contributing studies are ‘inferentially equivalent’, meaning that the constructs assessed are sufficiently comparable in their format, function or meaning. This requires consideration not just of whether data *can* be combined, but whether it *should* be combined. As noted above, some research teams choose to apply a common methodology across multiple studies or study centres in order to promote comparability of data at the point of collection; this is known as prospective harmonisation. In contrast, retrospective harmonisation refers to a process where efforts to foster comparability are initiated subsequent to data collection, such as through the pooling of data from studies that were hitherto distinct. Judgements relating to the potential for deriving harmonised variables across studies, and what format they might take, impact upon the types of research questions that can be addressed. In addition, these decisions influence how analytical results should be interpreted and applied by researchers, practitioners and policy-makers. Transparency in harmonisation methodology, therefore, is essential to evaluating the validity of results obtained from pooled data analyses and exploring their implications for subsequent research or policy. It also facilitates evidence synthesis and replication of analyses, but it is often lacking or insufficient. [19]

The International Children’s Accelerometry Database (ICAD) is a large, multi-country data pooling project, concerned with understanding the distribution, determinants and health impacts of objectively measured physical activity in young people (≤18 years). [31] ICAD draws together studies conducted in Europe, North and South America and Australia, all of which measured physical activity in young people using the Actigraph (Pensacola, FL) accelerometer. Given its scale and geographic diversity, ICAD is a potentially valuable resource to enhance our understanding of the correlates and determinants of physical activity and sedentary behaviour in young people. This evidence is essential to inform the design of effective behaviour change interventions, but much of the existing literature on this topic is drawn from single-country, cross-sectional studies that relied on self- or proxy reports of the outcome and addressed only a limited set of exposures. [8, 32–34] As detailed below, a key aim of the project to expand ICAD was to add more data on the personal, social and environmental factors that might influence children’s physical activity and sedentary behaviour in order the strengthen this evidence base.

The objective of this paper is to describe the data management and harmonisation methodology of ICAD and reflect upon the administrative, logistical and conceptual challenges that characterise work of this nature. More specifically, the paper aims to: 1) provide an overview of the development of ICAD and recent work to expand it; 2) summarise the methods for collating, cataloguing and managing ICAD data; 3) describe procedures for the harmonisation of non-accelerometer data (including examples); 4) discuss future directions for ICAD as a resource, including technical and operational considerations. Our primary focus is on the treatment of the non-accelerometer data in ICAD, as this has not been described previously in depth. This paper is complimentary to a previous publication describing the design and methods of ICAD, which focussed predominantly on the processing of accelerometer data. [31] It should be noted that all accelerometer data contained within ICAD were re-processed in 2015/16, with some amendments to the protocol used previously. Updated details on the processing of the accelerometer data is available from the ICAD website (http://www.mrc-epid.cam.ac.uk/research/studies/icad/).

## Methods

### ICAD – Background, oversight and access

A collaboration between the Medical Research Council (MRC) Epidemiology Unit and the universities of Bath and Bristol, ICAD was established in 2008 with funding from the UK National Prevention Research Initiative. Building upon the increasing use of accelerometry in physical activity research, ICAD was devised to enhance understanding in 3 key areas: 1) Levels and patterns of physical activity in children from diverse, social and geographic backgrounds; 2) social, cultural, ethnic and geographical determinants of physical activity; 3) dose-response relationships between components of physical activity and a range of health outcomes. Twenty studies were recruited to join ICAD and deposited data for processing between September 2008 and May 2010. All provided a signed agreement for the inclusion of study data in ICAD. The pooling strategy required all contributors to submit raw (unprocessed) accelerometer data and related non-accelerometer files, along with accompanying questionnaires and protocols, to a single location for processing and merging. As a minimum, partners were required to share their accelerometer data and information on participants’ sex, age, height and weight, but were free thereafter to submit as much or as little additional data as they wished. Background information and details on the processing of accelerometer data for this iteration of ICAD has been reported previously. [31]

Currently, day to day management and administration of ICAD is undertaken by the Working Group (AJA, UE, DWE, BHH, LBS, EMFvS), comprising representatives from the University of East Anglia, Loughborough University, the Norwegian School of Sport Sciences and the MRC Epidemiology Unit. Scientific oversight is provided by the Steering Committee, which comprises representatives from all contributing partners and the Working Group. The MRC Epidemiology Unit (University of Cambridge, UK) manages the database and data releases. Through a managed application process, ICAD data are available for use by any bona fide researcher. [35] Further details on the management of ICAD, contributing partners and the application process are available on the ICAD website (http://www.mrc-epid.cam.ac.uk/research/studies/icad/).

### ICAD 2 – Expanding the database

The first iteration of ICAD contained relatively little information on the personal, social and environmental factors that might interact to influence children’s activity. To address this limitation, and strengthen its capacity for the conduct of longitudinal analyses more generally, a project to expand ICAD was initiated in 2014. The expansion focussed on existing ICAD studies, who were invited to submit any additional waves of data that had been collected in their study and data on a broader range of personal, social and environmental characteristics. A shortlist of constructs that were considered potentially valuable additions to ICAD was prepared by the Working Group and subsequently circulated to a panel of invited experts (SJHB, STB, MCAP) and the Steering Committee for feedback, amendments and additions. Additional constructs requested for inclusion in ICAD are listed in Table 1. Constructs were defined in broad terms in order to encourage partners to share all potentially relevant variables on each construct.

**Table 1:** Social, behavioural and environmental variables added to ICAD database

Domain	Construct	Description
Home and family	Parental composition	Number of parents/guardians living at home.
	Internet access	Availability of internet in the family home.
	Number of televisions	Number of televisions in the family home.
	Garden	Access to a garden at home.
	Home location	City, County/State, Urban/Rural.
Physical activity – behaviour and correlates	Sport and organised physical activity	Child’s participation in sport or other organised physical activities.
	Physical activity self-efficacy	Child’s self-efficacy for engaging in or increasing physical activity.
	Parent support for physical activity	Mother/Father/Guardian support for child’s activity.
	Peer support for physical activity	Peer support for child’s activity.
	Parental physical activity	Mother/Father/Guardian physical activity level.
Sedentary behaviour – behaviour and correlates	TV in the bedroom	Presence of a television in child’s bedroom.
	Computer in the bedroom	Presence of a computer (inc. video games machine) in child’s bedroom.
	Sedentary behaviour self-efficacy	Child’s self-efficacy for reducing sedentary behaviours
	Parent support for sedentary behaviour	Mother/Father/Guardian support for child’s sedentary behaviour.
	Peer support for sedentary behaviour	Peer support for child’s sedentary behaviour.
	Parental television viewing	Mother/Father/Guardian time spent watching television.
	Parental computer use	Mother/Father/Guardian time spent using a computer.
School factors and other behaviours	Physical education	Child’s engagement in physical education at school.
	School start/end time	Start/end time of child’s school day.
	Sleep duration	Child’s sleep duration.
	Fruit and vegetables	Child’s consumption of fruits and vegetables.
	Sugar sweetened beverages	Child’s consumption of sugar sweetened beverages.
	Breakfast consumption	Whether or not child eats breakfast.

Only constructs added to ICAD as part of the expansion project are detailed here. A complete listing of all constructs available in ICAD is provided on the ICAD website

To facilitate data sharing, summary documents were prepared for each study, detailing what data had been submitted to ICAD at its inception. This document, along with details of the additional variables of interest and instructions for data transfer, was emailed to each study partner and a nominated data manager or co-investigator where appropriate. Partners were also requested to share all relevant supporting material, such as study protocols, standard operating procedures and questionnaires, to inform data cataloguing and harmonisation. Partners were under no obligation to share additional data, and were free to submit as much or as little as they felt appropriate. Data requests were circulated during the second half of 2014 through early 2015 and submission of new data accepted up to the end of 2015. Data cataloguing, processing and harmonisation was undertaken throughout 2016, with a new database released in spring 2017.

### Data management

We requested that accelerometer data were transferred as raw (unprocessed) files, in order that all accelerometer data could be reprocessed under a common protocol. No specification was made for the format of other accompanying data. Following initial checking and storage, two separate teams led on the management and processing of the accelerometer (BHH, LBS, UE, DWE) and non-accelerometer data (AJA, EMFvS). Accelerometer data were processed using KineSoft version 3.3.80 (KineSoft, Loughborough, United Kingdom). All non-accelerometer data were converted to STATA .dta ‘wide’ format master files (one row per participant). Where relevant, a prefix was added to variable names to indicate their time of assessment (e.g. W1_X = variable X at wave 1; W2_X = variable X at wave 2, etc.).

To inform harmonisation of the non-accelerometer data, a data dictionary was created for each study. Using a pre-prepared Microsoft Excel template, the following information was recorded for each variable: name, short label, detailed description, unit (e.g. cm, kg, mmHg), and format (e.g. continuous, categorical). The detailed description section included an extended description of the construct being assessed, the method of measurement, and category labels where appropriate. Identification numbers were assigned at the study and variable level to facilitate searching and corrections to be made. Each variable was assigned to a unique ‘variable group’, which identified the underlying construct to which it related. This was applied uniformly across all studies, enabling us to efficiently identify all variables that related to a particular characteristic. For example, all variables relating to child’s mode or duration of travel to school were tagged ‘School_travel’. Upon completion, each study template was uploaded to a single Microsoft Access database. The Access query function allowed for efficient and accurate extraction of specific batches of variables, identified using the variable groupings. Harmonised variables were created initially within each study master file and subsequently combined (appended) to create a single data file for data release (harmonisation procedures described below). The data dictionary of harmonised ICAD variables is accessible from the ICAD website (http://www.mrc-epid.cam.ac.uk/research/studies/icad/).

### ICAD harmonisation procedures for non-accelerometer data – An overview

In recognition of the time constraints of the Working Group and the likely personal preferences for harmonisation decisions of prospective users, an a-priori decision was made to create harmonised variables only for a sub-group of available constructs. The selection was based on specific research questions of interest to the Working Group and constructs which were considered to be most valuable to a wide range of prospective users of the updated database (e.g. as confounding variables). The shortlist included socio-demographic characteristics, a small number of candidate determinants of physical activity / sedentary behaviour and a range of anthropometric and metabolic factors. For each construct, information on relevant variables and methodology for all contributing studies was extracted from the data dictionary.

Data dictionary information was reviewed to establish consistencies (and inconsistencies) in the data across studies, and thus determine the potential for deriving harmonised variables. Although different from one construct to the next, key considerations here included: timeframe of assessment (e.g. proximity to accelerometer deployment, number of waves of assessment), data resolution (e.g. categorical vs. continuous), construct equivalence (pertinent for latent or multi-dimensional constructs), data source (indirect vs direct, objective vs subjective measures) and respondent (child- vs. parent-completed questionnaire). These considerations, amongst others, are discussed in the example below. Where a study collected data on a single construct from multiple sources (e.g. child *and* parent reported sex), an order of preference was established, along with procedures for dealing with missing or inconsistent data. As a general principle, we sought to create multiple harmonised variables for each construct, balancing the often competing demands of resolution and coverage (number of included studies). This enabled us to create higher resolution variables that made best use of detailed data where it was available and lower resolution variables that allowed for inclusion of the largest number of studies possible. This approach also allowed us to create harmonised variables to reflect the different components of multi-dimensional constructs, such as mode and duration of travel to school.

The complexity of the harmonisation process varied greatly dependent upon the particular characteristics of each construct. For anthropometric, metabolic and some demographic variables (e.g. age, sex), where there was general consistency in definition and assessment, harmonisation was conducted solely by the Working Group. For constructs that were deemed to be more conceptually or methodologically complex (e.g. ethnicity, car ownership, school travel, parent education) harmonised variables were created following an iterative process, with contributions from the Working Group, self-selected members of the Steering Committee (SK, JJP), and our panel of invited experts. The iterative process included four stages. First, one researcher proposed and derived an initial set of harmonised variables for each construct. Detailed documentation summarised the content and format of study-level data, reasons for exclusion of particular studies (or waves within studies) and any processing or recoding required to create the harmonised variables. Following circulation, all feedback was reviewed and amendments made to the format or procedure for creating harmonised variables as appropriate. Relevant documentation was updated and circulated to all parties for final review after which further amendments were made where necessary.

Detailed documentation was created to describe the data harmonisation process. This included information on the characteristics of the data provided by each study, a description of the harmonised variables created, lists of included/excluded studies/waves (along with relevant justification) and information about how multiple data sources and missing data were dealt with. Study specific notes were also produced, allowing for a more detailed explanation of unique design or methodology issues and how they were addressed. Lastly, tables were prepared to detail any study/wave-specific processing or recoding undertaken. Harmonised variables were created using algorithmic transformation or simple calibration methods. [19]. All harmonisation documentation is available on the ICAD website (http://www.mrc-epid.cam.ac.uk/research/studies/icad/data-harmonisation).

## Results

### Data harmonisation example - school travel mode and duration

The journey to school is a potentially important opportunity for children to accumulate physical activity. [36] Key research questions related to school travel include: “Do children who use active modes of travel to school accumulate more physical activity?” and “Is a change in travel mode associated with changes in physical activity?”. Information on school travel was requested when ICAD was first established but only a small number of studies (*n* = 8) provided this data. Additional data on school travel were requested, and received, as part of the expansion project. In this section, we outline the process and key considerations that informed the creation of three harmonised variables relating to school travel.

Fourteen studies (60%) provided data on one or more dimension of school travel (e.g. travel mode, duration, frequency). Seven studies provided data for two or more time points, and data were available for 25 study-waves in total. Information was collected by child-report in seven studies, by parent-report in four studies and three studies collected information by both child- and parent-report, either changing between waves or simultaneously from both within a single wave. In the latter case, parent-reported data were used preferentially as this was considered more likely to be reliable across all age ranges. Data referred to travel mode, frequency, duration and either the journey to or from school (or both), but few studies had information on all dimensions. Where information was available on both the journey to and from school, data on travel to school were used preferentially as this was reported most commonly across contributing studies.

Initial review highlighted the potential to create three harmonised variables; two regarding mode of travel and one describing duration of the journey (Table 2). Data from 11 studies (21 waves) were deemed suitable for inclusion in the categorical variable ICAD_SchoolTravel1. Four study-waves were excluded from this variable because the questionnaire items used referred only to walking or cycling to school, omitting other modes of travel such as car or bus. For these studies, we inferred that a response of no walking or cycling to school indicated that they used a non-active travel mode. Accordingly, all study-waves (*n* = 25) were included in the binary harmonised variable (ICAD_SchoolTravel2) which included only active / non-active travel mode categories. Eight studies provided information on duration of the journey to school, hence fewer data (8 studies, 13 waves) are included in the final harmonised variable (ICAD_SchoolTravel3).

**Table 2:** Overview of school travel harmonised variables

Name Description	Response categories	Summary statistics			
		Studies (n)	Waves (n studies)		
			1	2	3
ICAD_SchoolTravel1. Mode of travel to school.	Walk, Cycle, Public transport, Car, Other, Missing	11	4	4	3
ICAD_SchoolTravel2. Mode of travel to school	Active mode of travel, Other mode of travel, Missing	14	7	3	4
ICAD_SchoolTravel3. Duration of journey to school.	Less than or equal to 5 min, 6–15 min, More than 15 min, Missing	8	4	3	1

An overview of the source data and process for creating ICAD_SchoolTravel2 and ICAD_SchoolTravel3 is provided in Tables 3, 4, and 5 using illustrative data from the SPEEDY, KISS and Ballabeina studies. [37–39] The harmonised variables were created by collapsing categories in the source data or applying the appropriate thresholds to create categories from a continuous variable. For the SPEEDY study (waves 1 and 3), the questionnaire addressed school journey duration by walking and cycling separately but requested a combined estimate of journey duration if the participant travelled by bus or car. Therefore, responses for ICAD_SchoolTravel3 are provided only for those who indicated that they walked or cycled to school. In Ballabeina, information on the duration of the school journey was collected in two waves of assessment; however, the response categories used were not compatible with those selected for the harmonised variable. Therefore no data from this study were included in ICAD_SchoolTravel3. Complications of a comparable nature were encountered in other studies, including the use of questionnaires that allowed for the selection of multiple modes and frequencies of travel to school and the use of different questionnaires within sample subgroups. All such issues are discussed in the ‘study-specific notes’ section of accompanying documentation, available from the data harmonisation section of the ICAD website.

**Table 3:** Assessment of school travel mode and duration: Examples from three studies

	SPEEDY	KISS	Ballabeina
Respondent	Child-reported.	Parent-reported. Translated from German.	Parent-reported. Translated from French/German.
Item: Mode	How do you usually travel to school? Response options: Car; Bus/Train; Bicycle; On foot.	How does your child usually travel to school and how long does it take when following the most direct route (please name only the most common mode of transport). Response options: Walk; Bicycle/Scooter; Car; Bus/Train/Tram.	How does your child make it to school? Response options: Walk; Bicycle/Scooter; Bus/Tram; Car; Other.
Item: Duration	For the previous 7 days: Each time you did this (prompts: walk to school; cycle to school; travel to school by car / bus), how long did you normally do it for? Estimated as free text in minutes.	See above. Estimated as free text in minutes.	What is the duration of the trip (one way) to get to school? Response options: <10 min; 10–20 min; >20 min.
Item: Frequency	For the previous 7 days: How many times did you do this activity (prompts: walk to school; cycle to school; travel to school by car / bus). Response options: Never; Once; 2 to 3 times; 4 or more times.	N/A	N/A

For brevity, details are provided for data collected at study wave 1 only

**Table 4:** Overview of source data from three studies and harmonisation process for derivation of ICAD_SchoolTravel2

Source data			Harmonisation		
Study Wave	Variable(s): name(s), description	Summary	Category	Processing	Summary
SPEEDY Wave: 1	Variable name: W1_school_travel Mode of travel to school	Car, *n* = 923 Bus/train, *n* = 127 Bicycle, *n* = 189 On foot, *n* = 814 Missing, *n* = 11	Active mode	If [W1_school_travel] = Bicycle OR On foot	*n* = 1003
			Other mode	If [W1_school_travel] = Car OR Bus/train	*n* = 1050
			Missing	If [W1_school_travel] = Missing	n = 11
KISS Wave: 1	Variable name: a_schulweg_hin_sommer Mode of travel to school during summer	Walk, *n* = 392 Cycle/scooter, *n* = 51 Car, *n* = 7 Bus/train/tram, *n* = 3 Missing, *n* = 87	Active mode	If [a_schulweg_hin_sommer] = Walk OR Cycle/scooter	*n* = 443
			Other mode	If [a_schulweg_hin_sommer] = Car OR Bus/train/tram	n = 10
			Missing	If [a_schulweg_hin_sommer] = Missing	n = 87
Ballabeina Wave: 1	Variable name: W1_a_schulweg Mode of travel to school	Walk, *n* = 546 Cycle/scooter, n = 1 Bus/tram, n = 4 Car, *n* = 32 Other, *n* = 24 Missing, *n* = 62	Active mode	If [W1_a_schulweg] = Walk OR Cycle/scooter	*n* = 547
			Other mode	If [W1_a_schulweg] = Bus OR Car OR Other	*n* = 60
			Missing	If [W1_a_schulweg] = Missing	n = 62

For brevity, processing is summarised for data collected at study wave 1 only. Similar procedures were used for subsequent waves

**Table 5:** Overview of source data from three studies and harmonisation process for derivation of ICAD_SchoolTravel3

Source data			Harmonisation		
Study Wave	Variable(s): name(s), description	Summary	Category	Processing	Summary
SPEEDY* Wave: 1	Variable names: W1_a2acthrs_clean W1_a2actmins_clean W1_a3acthrs_clean W1_a3actmins_clean Duration of journey when walking or cycling to school	Continuous variables. Duration when walking in minutes per day Mean: 12.7 Median: 10 25th percentile: 5 75th percentile: 15 Range: 0, 60 Duration when cycling in minutes per day Mean: 11.2 Median: 10 25th percentile: 5 75th percentile: 15 Range: 0, 60	Less than 5 min	If duration of walking or cycling <=5	*n* = 356
			6–15 min	If duration of walking or cycling = 6–15 (inclusive)	*n* = 432
			More than 15 min	If duration of walking or cycling >15	*n* = 193
			Missing		*n* = 1083
KISS Wave: 1	Variable name: a_schulweg_hin_sommer_time Duration of journey to school	Continuous variable. Duration in minutes per day: Mean: 9.9 Median: 10 25th percentile: 5 75th percentile: 15 Range: 1, 30 Missing, *n* = 88	Less than 5 min	If [a_schulweg_hin_sommer_time] < =5	*n* = 153
			6–15 min	If [a_schulweg_hin_sommer_time] = 6–15 (inclusive)	*n* = 249
			More than 15 min	If [a_schulweg_hin_sommer_time] >15	*n* = 50
			Missing	If [a_schulweg_hin_sommer_time] = Missing	n = 88
Ballabeina Wave: 1	Variable name: W1_a_langeweg Duration of journey to school	Categorical variable: <10 min, *n* = 514 10–20 min, *n* = 82 >20 min, *n* = 10 Missing, *n* = 63	Response categories for this study captured different duration boundaries to those of the harmonised variable and could not be suitably collapsed or otherwise matched. Therefore, this study was not included in the harmonised variable.		

For brevity, processing is summarised for data collected at study wave 1 only. Similar procedures were used for subsequent waves. *The SPEEDY questionnaire did not allow for separate estimation of journey duration amongst those who travelled by bus/train or car to school. Therefore, only participants who indicated walk or cycle as their mode of travel were included in his variable

## Discussion

This paper provides background information and outlines the rationale and methodology for the expansion of a large, multi-study repository of accelerometer data in young people. ICAD remains unique within the field and the expansion work outlined herein serves to broaden its scope and facilitate the conduct of longitudinal analyses. For the benefit of ICAD users and those undertaking similar work, we sought to provide methodological transparency on our approach to data collation and harmonisation. Below we discuss our methods in the context of other data-pooling projects in the field of epidemiology more broadly, reflect upon some of the challenges encountered and consider future directions for ICAD.

### Logistical and methodological challenges of data pooling

ICAD is a large multi-partner, multi-country collaboration and is, therefore, subject to the same challenges of any such project, be it data pooling, primary data collection or otherwise. These include managing conflicting priorities amongst partners, maintaining effective and timely lines of communication, and managing large volumes of data. The ICAD approach to data pooling entailed submission of data to a single institution, with all subsequent data management, cataloguing and harmonisation undertaken by the Working Group. This approach was adopted to minimise the burden on individual partners and thus maximise their engagement with the project, but this placed significant burden on the Working Group. Some of the studies included in ICAD are historic or little used outside of the ICAD context, therefore there were sometimes challenges in obtaining information on the collection or derivation of particular variables. This limited the completeness of information that could be provided in the data dictionary for these studies.

As a data pooling methodology, collation into a centralised repository, as implemented in ICAD, is advantageous as it allows data to be analysed at the individual-level. [18] However, ethical issues and concerns over confidentiality and protection of intellectual property limit the application of this method and may dissuade, or even preclude, participation of some studies in projects of this kind. [22, 25, 26] Alternatives to centralised pooling, which allow study data to be retained within the host institution, may be more consistent with ethical requirements for some studies and help to allay fears over data security and confidentiality. In such cases, study-specific analyses may be undertaken by each study team, following an analysis plan prepared by the lead investigators. Study-level estimates are then submitted to the lead investigator where they are combined by meta-analysis. This approach, commonly used in genetic epidemiology, is potentially burdensome for each study investigator, as they are responsible for data harmonisation and analysis. Another option is to use a federated infrastructure, which allows for analysis at the individual level, undertaken by the lead investigator, whilst data are retained on local servers. [22, 23, 25, 26] This is achieved by the parallel analysis of individual study data, co-ordinated from a central computer over a secure internet connection (HTTPS). This approach represents perhaps the best combination of analytical flexibility and compliance with ethical and confidentiality issues currently available, though it requires a relatively complex technological infrastructure compared to other methods and is still under development. However, given its numerous advantages, elements of the federated approach may be appropriate for inclusion in future iterations of ICAD.

### Data harmonisation

A growing body of literature is emerging that deals with the ethical, methodological and technological issues that arise from data pooling and retrospective data harmonisation. [19, 21, 22, 29] A primary limitation of much previous work of this nature was a lack of methodological clarity, an issue which we have sought to address directly through this paper and related material on the ICAD website. For our initial data release of an expanded ICAD, we focussed on a subset of core variables for harmonisation and sought input from a range of subject experts. This proved extremely valuable with numerous amendments or additions made as a result of their feedback. Nonetheless, we recognise that others may contest the format or content of existing harmonised variables. Accordingly, we are keen to support other researchers in deriving their own harmonised variables and have developed a secure platform through which they can access raw study-level data to facilitate this. This system can also be used to create harmonised variables for the many constructs not currently included in the database, such as dietary behaviours and characteristics of the home and family. We have prepared a guidance document and template form to allow users to record the process of deriving new harmonised variables; this will be published and new variables uploaded to the database for further use.

### Future directions

To this point, work to expand ICAD has focussed upon collating more data from existing partners. As we consider further developments in the years to come, one avenue would be to recruit new partners into the consortium. Indeed our initial plans for expanding ICAD included the recruitment of new studies but as the project progressed it became apparent that this would not be possible within our preferred timeline and staffing capacity and this phase was put on hold. Upon release of the new database, discussions concerning the recruitment of new partners into ICAD will be reinstated. This discussion will include consideration of the scientific value of establishing new partnerships and how new studies would be identified and prioritised for inclusion, taking account of population representation (e.g. particular age groups, representation of low and middle-income countries) amongst other things. In addition, developments in activity assessment (such as wrist-based monitoring and the collection of raw acceleration data) mean there is a need to consider whether and how to incorporate studies that used other devices and/or body placements. This point notwithstanding, there remains a large number of existing studies that used methods compatible with ICAD (e.g. Actigraph, waist-worn monitors) that may be valuable additions to the database. Any future plans to expand ICAD would also require careful reflection on the administrative and technological approach to data collation and harmonisation, acknowledging the need to distribute the burden of work equitably amongst study personnel, ICAD users, the Working Group and support staff. There may be value in exploring other approaches to data storage and the steps that can be taken to facilitate accessible and efficient data harmonisation and analysis by ICAD users. We welcome expressions of interest from principal investigators interested in joining ICAD and insights from a methodological or technological perspective that would feed into our discussions on this topic.

### Strengths and limitations

Through this paper and material posted on the ICAD website, we have described our methods of harmonising data from multiple studies on the correlates of physical activity in young people. In so doing, we sought to address a well-recognised limitation of much previous work of this nature; that is, a lack of transparency in data processing and harmonisation. [19] We undertook extensive data cataloguing at the study-level and of harmonised variables, enabling ICAD users and the wider research community to fully understand the data available for analysis and to enable them to derive new harmonised variables where necessary. The format and content of existing variables was determined iteratively, with input from the ICAD Steering Committee and invited subject experts. The following limitations are acknowledged. Firstly, the burden of data preparation and cataloguing (for both accelerometer and non-accelerometer data) fell heavily on the ICAD Working Group. Whilst beneficial in terms of consistency and partner engagement, this model would not be sustainable in future developments of the database and alternative approaches, such as requesting that partners undertake some of this preparatory work themselves, will need to be explored. We also acknowledge that despite our rigorous approach to data harmonisation, these decisions are subjective and other researchers may disagree with the content of existing variables. In such cases, under secure data conditions, we will enable researchers to access study-level data, allowing them to create new harmonised variables to their preferred specification.

ICAD is currently the largest existing repository of accelerometer data in young people, with data available for approximately 30,000 individuals between the ages of 3 and 18 years. The expanded database includes longitudinal physical activity data from 13 studies, greatly improving its capacity for the conduct of longitudinal analyses relative to its predecessor. Alongside the accelerometer data, information is available for a range of demographic, anthropometric, metabolic, behavioural and environmental characteristics. Limitations of ICAD include the relative under-representation of certain age groups (<8 years) and participants from low- and middle-income countries. The majority of partner studies do not comprise nationally representative samples, thus findings of physical activity prevalence for example, should be generalised with caution. Lastly, although every effort was made to obtain information on study protocols and instrumentation, we were unable to capture any verbal instructions or guidance provided to participants by the data collection teams at the point of assessment. Such instructions, however, are likely to have been uniform within each study and of minimal influence of participant responses.

### Recommendations

Herein we provide general recommendations to facilitate the process of pooling and harmonising epidemiological data. These will likely be relevant to a range of research settings, beyond the specific population and topic addressed in this paper.

1. Consider the potential for data sharing at the point of project initiation and, where appropriate, request support from funders to facilitate this.
2. Ensure that participants are informed of, and consent to, the possibility of their data being used beyond the original study.
3. Potential for data sharing and harmonisation may inform instrument selection and application. High resolution data are more amenable to retrospective harmonisation than low resolution data.
4. Establish structured and transparent data management processes. Include data management expertise in the project team and, where possible, retain for the entire duration of the project.
5. Ensure that study administration is detailed and complete. This may include a protocol of recruitment and measurement procedures, and the preparation of ‘standard operating procedures’ (SOPs), data dictionaries and syntax libraries of data management and cleaning processes.
6. Data pooling terms and conditions should be outlined in formal data sharing and user agreements, and agreed by all partners.

## Conclusion

The ICAD expansion project demonstrates that large-scale pooling of data related to young people’s physical activity, and its associated correlates and health outcomes, is feasible. A rigorous and transparent process of retrospective data harmonisation facilitates the conduct of pooled analyses. This work has greatly enhanced capacity for the conduct of longitudinal analyses and exploration of the determinants of physical activity across childhood and adolescence in ICAD. Details of our methodology for data collation and harmonisation are provided to assist those undertaking similar projects, aid the analysis and interpretation of data and facilitate widespread use of this resource.
